# Effects of Iron and Vitamin A Levels on Pregnant Women and Birth Outcomes: Complex Relationships Untangled Using a Birth Cohort Study in Uganda

**DOI:** 10.1007/s10995-022-03387-5

**Published:** 2022-03-03

**Authors:** Julieta Mezzano, Grace Namirembe, Lynne M. Ausman, Elizabeth Marino-Costello, Robin Shrestha, Juergen Erhardt, Patrick Webb, Shibani Ghosh

**Affiliations:** 1 Friedman School of Nutrition Science and Policy, Tufts University, 150 Harrison Avenue, Boston, MA 02111 USA; 2 VitMin Lab, Kastanienweg 5, 77731 Willstaett, Germany

**Keywords:** Vitamin A, Iron, Birth outcomes, Pregnancy, Uganda

## Abstract

**Introduction:**

Women and infants are among the most vulnerable groups for micronutrient deficiencies. Pregnancy micronutrient status can affect birth outcomes and subsequent infants’ growth.

**Methods:**

We determined the relationship between maternal iron and vitamin A status at delivery using several biomarkers (ferritin, soluble transferrin receptor [sTFR], body iron stores [BIS], hemoglobin and retinol binding protein [RBP]) and birth outcomes (body weight, Z-scores, head circumference, small-for-gestational-age and preterm birth) in rural Uganda. We investigated women who had serum results at the point of delivery and paired them to their infants at birth (n = 1244). We employed multivariable linear and logistic regression, adjusting for clustering at the subcounty level to determine the relationship between maternal micronutrients and birth outcomes.

**Results:**

After adjusting for relevant factors, we found that maternal iron status (ferritin and BIS) and anemia (hemoglobin) were not significantly associated with the assessed birth outcomes. However, there was a significant association between serum sTFR and preterm births (AOR: 0.67; 95% CI 0.48–0.94). For Vitamin A, we observed a significant positive association between RBP and length-for-age (LAZ) at birth (β = 0.12, *p* < 0.030).

**Discussion:**

These findings indicate that the relationship between maternal iron status and birth outcomes needs to be further investigated, because depending on the biomarker used the associations were either in favor of an adverse birth outcome or not significant. Additionally, they confirm that higher maternal RBP levels could be beneficial for birth outcomes.

*Clinicaltrials.gov* as NCT04233944.

**Supplementary Information:**

The online version contains supplementary material available at 10.1007/s10995-022-03387-5.

## Significance

There is conflicting evidence of the different types of biomarkers used to assess maternal micronutrients status (in this case iron and vitamin A) and their relationship with birth outcomes. Our study aims to address this question by using the three major indicators of iron status (ferritin, sTFR and BIS) and RBP for vitamin A status, in a relatively large sample of mothers in rural Uganda. In addition, to achieve more precise estimates, we correct for inflammation using C-Reactive Protein (CRP) and α-1 acid glycoprotein (AGP) as per suggested in the most recent literature.

## Introduction

Women and infants are among the most vulnerable groups for micronutrient deficiencies (CDC, 2019). Iron and vitamin A have been identified globally as two critical micronutrients, particularly during the period of pregnancy and early life (Gernand et al., 2016). In 2011, anemia affected almost half of the children under five years of age and more than a third of pregnant women, worldwide (CDC, 2019). Vitamin A deficiency alone affected one out of six pregnant women globally, and was responsible for almost 6% of child deaths under the age of 5 years in Africa (WHO, 2011a).

Difficulties arise when assessing micronutrient deficiencies in countries where a high burden of infection exists because biomarkers used to measure them are triggered by inflammation (Lynch et al., 2018). The Biomarkers Reflecting Inflammation and Nutritional Determinants of Anemia (BRINDA) project, has therefore highlighted the importance of adjusting biomarkers of Vitamin A and iron for inflammation and for malaria status (Namaste et al., 2017).

During the last decade, several studies have analyzed relationships between maternal micronutrient status during pregnancy and birth outcomes. A recent Lancet study reported that multiple micronutrient supplementation during pregnancy yielded favorable birth outcomes in infants born to undernourished and anemic women (Smith et al., 2017). For iron, however, the relationship between maternal iron status and birth outcomes remains unclear since recent studies showed conflicting evidence. Although most studies have shown a positive association between iron-replete mothers and birth outcomes (Alwan et al., 2015; Rahman et al., 2016; Srour, 2018; Yang et al., 2017), some have reported a negative association (Fowkes et al., 2018; Hsu et al., 2013; Yuan et al., 2019), or in some cases, no significant association (Dewey & Oaks, 2017; Samimi et al., 2012; Zhang et al., 2018). Even where studies found a positive association between maternal iron status and birth outcomes, this was true for some outcomes but not others. For example, one review reported that iron-deficiency anemia (IDA) was associated with an increased risk of preterm delivery, low birth weight (LBW), and maternal and child mortality (Lynch et al., 2018). However, two other studies (Fowkes et al., 2018; Yuan et al., 2019) reported that iron deficiency and anemia are protective against adverse birth outcomes in areas with a high infection burden. The most recent systematic review of maternal iron status and birth outcomes (Dewey & Oaks, 2017) also showed mixed evidence. It is worth noting that the above studies used different biomarkers to assess the micronutrients and only some adjusted for inflammation. Thus, research is needed to evaluate if the higher risk of adverse outcomes associated with higher iron levels is mainly due to underlying/co-existing inflammation or infection, rather than iron status. This paper is an attempt to contribute to that evidence gap by using different biomarkers corrected for inflammation with the latest recommended approach in the literature (BRINDA) thus obtaining more accurate micronutrients estimates.

Vitamin A deficiency during pregnancy is also associated with poor birth outcomes (Christian et al., 2013). A study in India assessed the effects of vitamin A deficiency during pregnancy in women (Radhika et al., 2002) and found that low serum retinol was associated with an increased risk of preterm delivery and maternal anemia. In another study, vitamin A supplementation during pregnancy did not have a significant overall effect on birth outcomes but it was protective of LBW in HIV-positive women (Thorne-Lyman & Fawzi, 2012). As for iron deficiency, the commonly used biomarkers to estimate vitamin A deficiency (serum retinol and retinol binding protein [RBP]) become temporarily altered with high levels of inflammation, potentially overestimating its prevalence.

The objective of this study was to determine the relationship between maternal iron and vitamin A biomarkers (ferritin, soluble transferrin receptor [sTFR], body iron stores [BIS], hemoglobin and RBP) and birth outcomes [body weight, weight for age (WAZ), length for age (LAZ) and weight for length (WLZ) Z-scores, head circumference, small-for-gestational-age and preterm birth] after adjusting for relevant factors in rural Uganda. A second objective was to describe the prevalence of iron and vitamin A deficiencies, anemia, and IDA in this population of mothers with and without adjusting for inflammation using the BRINDA correction.

## Methods

The Uganda Birth cohort (UBC) study was a longitudinal prospective birth cohort of pregnant women aged 15–49 years from rural North and South-Western subcounties (geographic administrative units) of Uganda. Funded by the United States Agency for International Development (USAID) the UBC was conducted between 2014 and 2016. The aim was to intensively study pregnant women, their children and households to understand the impact of agriculture, nutrition and health interventions provided by the Uganda Community Connector project (CC) (USAID, 2015). Pregnant women were recruited using a pregnancy surveillance system consisting of local village health teams (VHTs) through home visits, based on the eligibility criteria. Pregnancy was ascertained using a urine pregnancy test. Pregnant women of all gestational ages were recruited on a rolling basis over a span of 12 months.

Of the women enrolled, data from 4949 were available by Visit 1. Women and their infants were followed throughout the first year of the child’s life. Data were collected at enrollment, birth (Visit 3), and at 3 (Visit 4), 6 (Visit 5), 9 (visit 6) and 12 (Visit 7) months from the date of delivery (see Fig. 3 in Appendix). Data on diets, anthropometric measurements, diseases, vaccinations, income and expenditure, among others, were collected with in-person interviews by trained enumerators in each subcounty (Madzorera et al., 2021).

All subjects gave informed consent for inclusion in the study before participation. The study was conducted in accordance with the Declaration of Helsinki. The protocol was approved by the Higher Degrees, Research and Ethics Committee (HDREC) of Makerere University School of Public Health, and the Institutional Review Board (IRB) from both Tufts Health Sciences and Harvard T.H. Chan School of Public Health. The UBC study was registered at clinicaltrials.gov as NCT04233944.

**Fig. 1:**
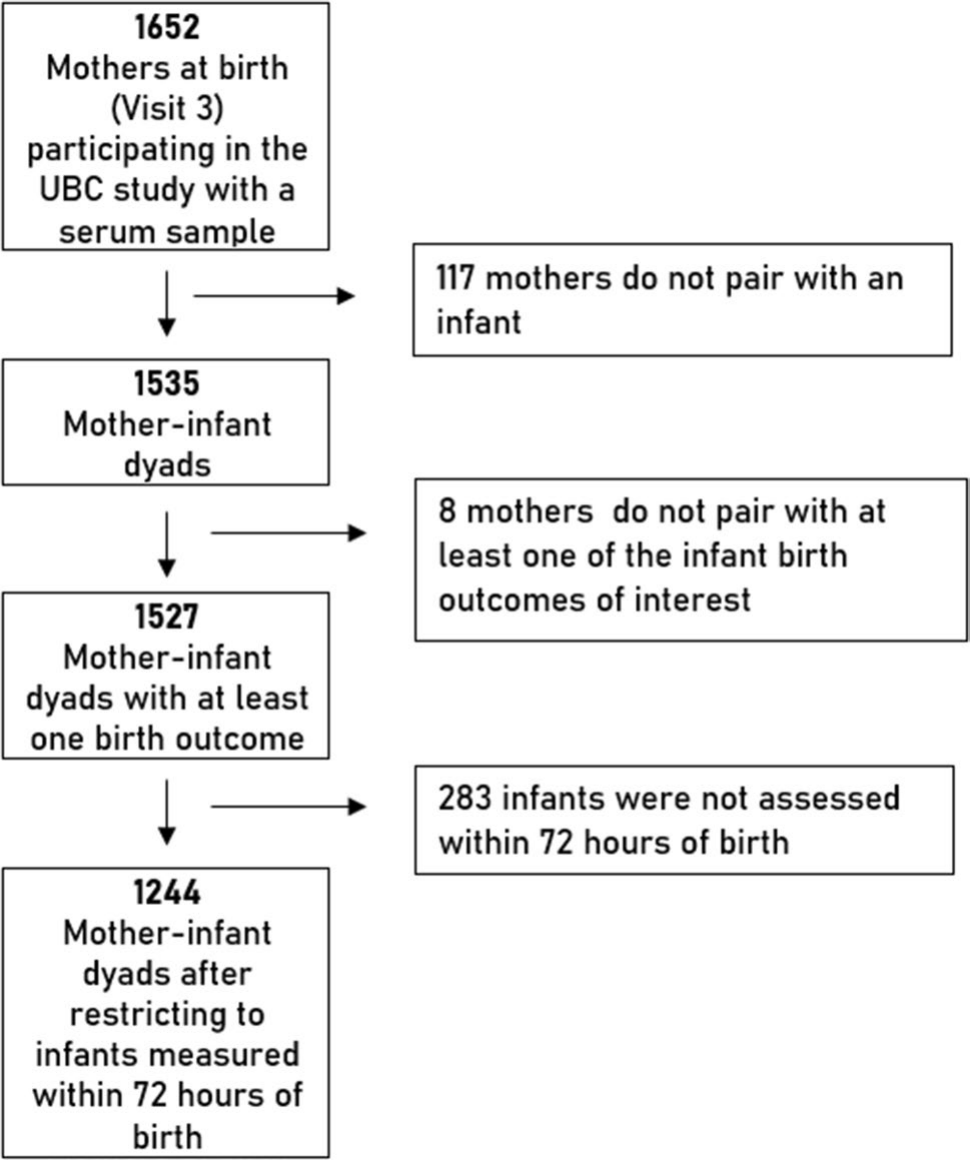
Flowchart showing the subset of study participants from the UBC study. Multiple births were not excluded from the UBC study but were omitted from the analyses

In this paper, we investigated a subsample of women who had serum sample results at the point of delivery and paired them to their infants measured within 72 h of birth (n = 1244) to assess the relationship between biomarkers and birth outcomes (Fig. 1). Refer to Table 7 in the Appendix for the number of pregnancies per subcounty.

Women’s venous blood samples (3–5 mL) were drawn at the point of delivery (38–42 weeks of gestational age), and the resultant serum was frozen and shipped to US and Germany for processing, distribution and analysis. The VitMin Lab in Germany analyzed ferritin (FER), sTFR, RBP, and inflammatory markers C-Reactive Protein (CRP) and α-1 acid glycoprotein (AGP) using a sandwich enzyme-linked immunosorbent assay (Erhardt et al., 2004).

BIS were calculated as the ratio of sTFR/FER using the formula: body iron (mg/kg) =  − [log(sTFR/FER ratio) − 2.8229]/0.1207 (Cook et al., 2003). Hemoglobin (Hgb) levels were measured using a HemoCue test by trained nurses to assess anemia status, and resultant values were adjusted for altitude (WHO, 2011b).

The measurement of ferritin as a proxy for iron stores is a widely used method and it is recommended to report iron status of populations. Along with sTFR, and the calculation of BIS, they are the best-suited parameters to assess iron status (Cook et al., 2003). The marker sTFR correlates well with ferritin, increasing when iron deficiency exists (Erhardt et al., 2004).

Although the measurement of liver reserves of vitamin A is the gold standard method for estimating vitamin A status, it is unrealistic to perform biopsies on live people. Thus, circulating retinol assessment is the most commonly used technique (Tanumihardjo et al., 2016). The assessment of serum RBP as a proxy for vitamin A is also widely used since circulating retinol is carried on RBP and transthyretin in a very specific complex. In addition, the assay for RBP is straightforward, less expensive and RBP is highly correlated with retinol concentrations (Tanumihardjo et al., 2016).

Given the BRINDA recommendation, in this analysis, FER and sTFR were corrected for inflammation (CRP and AGP) using their regression correction approach (Namaste et al., 2017). As adjustment of RBP values for inflammation have shown inconsistent results in adult women; we did not adjust maternal RBP values in this paper (Larson et al., 2018). The following cutoffs were applied to define iron deficiency and anemia: ferritin < 15 µg/L, sTFR > 8.3 mg/L, BIS < 0 mg/kg, and Hgb < 11 g/dL; vitamin A deficiency: RBP < 0.7 µmol/L; and inflammation: CRP > 5 mg/L, AGP > 1 g/L (Cook et al., 2003; Erhardt et al., 2004; Namaste et al., 2017; WHO, 2011b).

The outcomes assessed in this paper include birth weight, LBW, gestational age, small-for-gestational-age (SGA), preterm births, WAZ, LAZ, WLZ and head circumference. Birth weight (kg) was measured using a Seca 874 digital scale (accuracy ± 50 g) and calculated as the mean of three repeated measurements on each infant (Seca Corporation). Length was measured using a measuring board (accuracy 0.1 cm) (Infant/Child/Adult ShorrBoard®). LBW was defined as the percentage of infants with birth weight lower than 2500 g. Gestational age (days) was calculated as the date from last menstrual period to delivery date. Implausible gestational ages (< 168 or > 315 days and negative values) were excluded from analyses (n = 150). Two outcome variables were derived from gestational age: preterm birth and SGA. A preterm birth was defined as gestational age < 259 days (Howson et al., 2012). SGA was defined as infants who were small for gestational age and sex (weight centiles below the 10th percentile) using INTERGROWTH-21 standards.

Standardized anthropometric indices were computed using WHO child growth standards. Z- scores (WAZ, LAZ and WLZ) were calculated using infant’s age, weight and length variables at birth. Birth lengths less than 45 cm were excluded from the WLZ calculation. Other exclusions included WAZ <  − 6 or > 5, WLZ <  − 5 or > 5, and LAZ <  − 6 or > 6. Stunting, underweight and wasting were defined as LAZ, WAZ and WLZ less than − 2 standard deviations respectively. Head circumference (cm) was measured using a tape and computed as a mean of three different measurements on each child at birth.

The primary exposure variables included ferritin, sTFR, BIS, hemoglobin and RBP. Other covariates included Wealth Index, food security as measured by the Household Food Insecurity Access Scale (HFIAS) (Coates et al., 2017), Minimum Dietary Diversity for Women (MDD-W), parity, maternal education, maternal height, maternal mid-upper arm circumference (MUAC), participation in CC project and infant’s sex. Parity was defined as the number of live-born infants the mother ever had. Since parity and age of the mother were correlated, the latter was excluded from multivariable analyses. MDD-W was assessed during pregnancy and was met if the woman reported consuming five or more different food categories in the last 24 h. Maternal height (cm) was computed as the mean of three measurements on the same individual, recorded to the nearest 0.1 cm (ShorrBoard®). Women’s MUAC, obtained using measuring tape at the point of delivery, was used instead of weight or BMI. Membership in the CC project was accounted for in multivariate analyses as a potential confounder because the interventions received by participants may have had an effect on both micronutrient uptake and birth outcomes.

The relationship between maternal iron and vitamin A status with birth outcomes was assessed using multivariable linear regression for continuous outcomes and multivariable logistic regression for dichotomous outcomes, adjusting for clustering within subcounties. Variables that were significant at the *p* < 0.1 level in bivariate analyses were included as potential confounders in multivariable analyses. Univariate diagnostic plots showed that ferritin and sTFR were right-skewed and hence they were log-transformed; RBP was normally distributed and did not require transformation. All analyses were performed using SAS software, version 9.4.

## Results

### Maternal Characteristics

The sample characteristics for mothers participating in this substudy are presented in Table 1. Approximately half the women came from the Northern and the rest from the South-Western regions of Uganda. Women’s age ranged between 16 to 47 years [mean (± SD) 26.9 years (± 6.2)]. Their mean (± SD) height and weight was 159.2 cm (± 6.0) and 58.1 kg (± 8.1), respectively. The mean gestational age of women during the first visit was 186.3 days (± 57.7). On average, women completed five years of school, and the majority (95%) reported being married or cohabiting. The median household size consisted of five members with women having on average three children. Almost 95% of the household heads were male.

**Table 1:** Household and maternal characteristics during pregnancy

Characteristics	n (%)	Mean ± SD
Age (years)	1213	26.92 ± 6.21
Mother’s education (years)	1225	5.28 ± 3.02
Height (cm)	1241	159.23 ± 6.03
Weight (kg)	1240	58.08 ± 8.10
BMI (kg/m^2^)	1240	22.91 ± 3.00
MUAC (cm)^a^	1240	26.16 ± 2.40
Parity (number of infants)	987	3.13 ± 2.10
Gestational age (days)^b^	1194	186.25 ± 57.66
Household size (number of members)	1215	4.84 ± 2.44
Iron supplementation frequency (days)	1171	49.03 ± 32.22
IPT frequency (times)^c^	1018	1.72 ± 0.77
Region (%)	1225	
North	641 (52.33)	–
Southwest	584 (47.67)	–
Household head sex (%)	1225	
Male	1157 (94.45)	–
Marital status (%)	1225	
Married/cohabiting	1167 (95.27)	–
Wealth index (%)	1221	
1-Poorest	247 (20.23)	–
2-Poorer	258 (21.13)	–
3-Middle	249 (20.39)	–
4-Richer	249 (20.39)	–
5-Richest	218 (17.85)	–
HFIAS (%)^d^	1224	
1-Food secure	447 (36.52)	–
Vit. A rich food intake (plant) (%)	479 (39.10)	–
Vit. A rich food intake (animal) (%)	142 (11.59)	–
Iron rich food intake (heme) (%)	270 (22.04)	–
Iron rich food intake (non-heme) (%)	1147 (93.63)	–
MDD-W (%)^e^	1225	
Consumption of ≥ 5 food categories	210 (17.14)	–
Iron suppl. during pregnancy (%)	1244	
Yes	1171 (94.13)	–
Malaria (%)	1187	
Positive	65 (5.48)	–
Anti-malaria treatment (%)	1244	
Yes	1021 (82.07)	–

^a^Mid-Upper Arm Circumference

^b^Woman’s gestational age at first visit

^c^Intermittent Preventive Treatment

^d^Household Food Insecurity and Access Scale

^e^Minimum Dietary Diversity for Women

With respect to diets, only 17% of women met the MDD-W and 63% of the households were classified as food insecure. 39% of women reported consuming vitamin-A-rich plant foods, while only 11% vitamin-A-rich animal foods. Only 22% of the women reported consuming iron from heme sources, while the majority, 94%, from non-heme sources. Another source of iron was through supplementation: 94% of women reported taking iron-folate supplements during pregnancy but the mean duration of intake was only 49 days. Daily oral supplementation of iron (30–60 mg) and folic acid tablets is recommended in Uganda throughout pregnancy (Kiwanuka et al., 2017). Only 6% of the women tested positive for malaria and almost 82% reported receiving intermittent preventive treatment (IPT) against malaria.

### Infants’ Characteristics

The sample characteristics for infants participating in this substudy are detailed in Table 2. The mean (± SD) age of the infants at measurement was 1.6 days (± 1.0). Forty-nine percent were males. The mean ± (SD) birth weight and length of the infants was 3.3 kg (± 0.5) and 47.6 cm (± 3.3), respectively. About 4% had LBW and 91% fell within the normal birth weight category. Given the low prevalence of LBW, we did not include this variable as an outcome in the multivariable analyses. The mean (± SD) head circumference was 35.6 cm (± 1.7). However, the minimum value observed was 30.7 cm, indicative of microcephaly (CDC definition).

**Table 2:** Infants’ sample characteristics

Characteristics	n (%)	Mean ± SD	Median	Min	Max
Age (days)	1244	1.58 ± 0.97	2.00	0.00	3.00
Birth weight (kg)	1244	3.26 ± 0.49	3.20	1.50	6.07
Birth length (cm)	1244	47.57 ± 3.33	48.00	36.83	60.00
Head circumference (cm)	569	35.56 ± 1.66	35.30	30.70	43.10
Weight-for-age Z-score	1239	− 0.12 ± 1.00	− 0.10	− 4.47	4.91
Length-for-age Z-score	1233	− 1.00 ± 1.73	− 0.78	− 5.9	5.34
Weight-for-length Z-score	1006	0.54 ± 1.74	0.57	− 4.86	4.97
BMI Z-score	1215	0.60 ± 1.65	0.53	− 4.87	4.95
Gestational age (days)	1108	274.56 ± 21.71	277.00	168.00	315.00
Sex (%)	1240				
Male	602 (48.6)	–	–	–	–
Underweight (%)	30 (2.42)	–	–	–	–
Stunted (%)	325 (26.2)	–	–	–	–
Wasted (%)	80 (7.8)	–	–	–	–
Term birth (%)	1108	–	–	–	–
Pre-term	216 (19.49)	–	–	–	–
Full term	892 (80.51)	–	–	–	–
Birth weight categories (%)	1244				
Low birth weight	44 (3.5)	–	–	–	–
Normal birth weight	1134 (91.2)	–	–	–	–
High birth weight	66 (5.3)	–	–	–	–
Small for Gestational Age (%)	136 (13.6)	–	–	–	–

Measurements taken at birth

The prevalence of stunted, wasted, and underweight infants was 26.2%, 7.8% and 2.4%, respectively. The mean (± SD) standardized scores for WAZ (underweight), LAZ (stunting) and WLZ (wasting) were − 0.1 (± 1.1), − 1.0 (± 1.7), and 0.6 (± 1.7), respectively. The mean (± SD) gestational age at birth was 274 days (± 22) and 14% of the infants were born SGA. About 20% of all infants were born preterm. The mean (± SD) gestational age of those born preterm was 240.8 days (± 16.4) and at term, 282.7 days (± 13.3). Their range of gestational ages was 168–258 days and 259–315 days, respectively.

### Maternal Micronutrient Status

Table 3 shows the biomarkers levels of maternal iron, vitamin A and inflammation (AGP and CRP). The mean (± SD) ferritin and sTFR levels corrected for inflammation were 48.9 µg/L (± 30.1) and 7.3 mg/L (± 4.2), respectively. Mean (± SD) maternal Hgb levels (g/dL) were within normal ranges with a slight increase from 12.1 (± 1.5) during pregnancy to 13.0 (± 1.6) at delivery. However, minimum values showed that some women had severe anemia (5.50 and 7.00 g/dL during pregnancy and birth). For vitamin A, the mean (± SD) RBP was 2.0 µmol/L (± 0.8) and for AGP and CRP were 1.07 g/L (± 0.58) and 7.73 mg/L (± 17.87), respectively. The prevalence of women experiencing high inflammation (CRP > 5 mg/L and/or AGP > 1 g/L) was 56.2% (n = 699).

**Table 3:** Maternal micronutrient and inflammation biomarkers at parturition

Variable	n	Mean	SD	Median	Min.	Max.
Ferritin (corrected)^a^ (µg/L)	1244	48.90	30.13	42.73	3.38	176.88
Ferritin (µg/L)	1244	67.14	42.64	56.95	3.59	327.34
sTFR (corrected)^a^ (mg/L)	1244	7.25	4.18	6.02	2.05	45.20
sTFR (mg/L)	1244	7.80	4.92	6.35	1.67	49.74
Hemoglobin at enrollment^b^ (g/dL)	1109	12.10	1.45	12.20	5.50	18.40
Hemoglobin at birth^b^ (g/dL)	1186	13.00	1.61	13.10	7.00	17.80
RBP^c^ (µmol/L)	1244	1.97	0.77	1.84	0.41	4.00
AGP (g/L)	1244	1.07	0.58	0.93	0.13	4.57
CRP (mg/L)	1244	7.73	17.87	1.90	0.01	277.11
Albumin (g/dL)	1209	3.78	0.80	3.85	1.16	6.86

^a^Correction for inflammation using BRINDA Coefficient Regression method

^b^Adjusted for altitude

^c^BRINDA adjustment for inflammation not recommended in mothers or women of reproductive age

The prevalence of iron and vitamin A deficiencies and anemia in Ugandan mothers is depicted in Table 4. Adjustment for inflammation (BRINDA), increased the measure of depleted iron stores of ferritin from 7 to 12%, and decreased iron-deficient erythropoiesis (sTFR) from 27 to 22%. The prevalence of tissue iron deficiency (BIS < 0 mg/kg) using corrected sTFR and ferritin was 10% compared to 8% using uncorrected markers. The prevalence of altitude-adjusted anemia was 14% and 5% of the women had IDA.

**Table 4:** Prevalence of Iron and Vitamin A deficiencies and Anemia in Ugandan mothers (n = 1244, at birth)

Parameter of deficiency	Prevalence (%) (95% CI)	
	Uncorrected	Corrected for inflammation*
Iron depleted stores (FER)^a^	7.4% (6.0, 8.9)	12.3% (10.5, 14.3)*
Iron deficient erythropoiesis (sTFR)^b^	26.7% (24.3, 29.2)	21.6% (19.4, 24.0)*
Body iron stores (BIS)^c^	8.0% (6.6, 9.7)	10.5% (8.8, 12.3)^#^
Anemia (Hgb)^d^	11.0% (9.2, 12.9)	13.8% (11.9, 15.9)^^^
Iron deficiency anemia (IDA)^e^	–	4.5% (3.4, 5.8)
Vitamin A deficiency (RBP < 0.7 µmol/L)	1.5% (0.9, 2.4)	N/A^$^
Vitamin A deficiency (RBP < 0.83 µmol/L)^f^	3.1% (2.2, 4.3)	N/A^$^
Moderate vitamin A deficiency (RBP < 1.17 µmol/L)^f^	12.2% (10.5, 14.2)	N/A^$^

^a^Ferritin < 15 ug/L

^b^sTFR > 8.3 mg/L

^c^BIS < 0 mg/kg

^d^Hgb < 11 g/dL

^e^Having both Iron depleted stores and anemia

^f^using specific cut-offs for RBP instead of retinol

*Correction for inflammation using BRINDA Coefficient Regression method

^#^Using log of BRINDA-adjusted sTFR/FER

^$^BRINDA adjustment for inflammation for vitamin A not recommended in mothers or women of reproductive age

^^^Adjusted for altitude

To describe RBP values as a proxy for vitamin A deficiency, we used the recommended cut-off for retinol (RBP < 0.7 µmol/L) (WHO, 2011c). Some studies, however, have proposed different cut-offs specifically for RBP and pregnant women (Engle-Stone et al., 2011). Table 4 shows how vitamin A prevalence estimates differ based on the limits defined, ranging from 2 to 12%.

### Association Between Maternal Micronutrient Status and Birth Outcomes

Results for the associations between maternal iron and vitamin A biomarkers and birth outcomes are shown in Tables 5 and 6.

**Table 5:** Bivariate and multivariable analyses of maternal iron biomarkers and birth outcomes

	Unadjusted model			Adjusted model		
Ferritin (µg/L)^#^ & birth outcomes						
	n	*β* estimate (SE)	*p* value	n	*β* estimate (SE)	*p* value
Birth weight (kg)^a^	1244	− 0.03 (0.02)	0.153	1077	− 0.03 (0.02)	0.126
Weight-for-age Z-score^a^	1240	− 0.05 (0.04)	0.196	1076	− 0.05 (0.04)	0.175
Length-for-age Z-score^b^	1240	− 0.01 (0.07)	0.874	1072	− 0.07 (0.04)	0.132
Weight for length Z-score^c^	1020	− 0.03 (0.08)	0.722	867	0.11 (0.06)	0.081
Head circumference (cm)^d^	569	0.06 (0.10)	0.559	461	0.03 (0.11)	0.802
	n	OR (95% CI)		n	OR (95% CI)	
Small for gestational age^e^	1001	0.97 (0.76–1.24)	–	915	0.98 (0.72–1.34)	–
Preterm^f^	1403	1.11 (0.93–1.32)	–	1018	1.24 (0.92–1.65)	–

sTFR (mg/L)^#^ & birth outcomes						
	n	*β* estimate (SE)	*p* value	n	*β* estimate (SE)	*p* value
Birth weight (kg)^a^	1244	0.03 (0.03)	0.371	1077	− 0.02 (0.04)	0.662
Weight-for-age Z-score^a^	1240	0.06 (0.07)	0.375	1076	− 0.02 (0.07)	0.767
Length-for-age Z-score^b^	1240	0.17 (0.11)	0.126	1072	0.10 (0.14)	0.484
Weight for length Z-score^c^	1020	0.07 (0.13)	0.615	867	0.03 (0.13)	0.829
Head circumference (cm)^d^	569	0.16 (0.16)	0.309	461	− 0.03 (0.17)	0.868
	n	OR (95% CI)		n	OR (95% CI)	
Small for gestational age^e^	1001	0.75 (0.49–1.15)	–	915	0.96 (0.59–1.59)	–
Preterm^f^	1403	0.86 (0.64–1.16)	–	1018	0.67 (0.48–0.94)*	–

BIS (mg/kg)^$^ & birth outcomes						
	n	*β* estimate (SE)	*p* value	n	*β* estimate (SE)	*p* value
Birth weight (kg)^a^	1243	− 0.01 (0.00)	0.141	1077	− 0.00 (0.00)	0.380
Weight-for-age Z-score^a^	1239	− 0.01 (0.01)	0.171	1076	− 0.00 (0.01)	0.384
Length-for-age Z-score^b^	1239	− 0.01 (0.01)	0.419	1072	− 0.02 (0.01)	0.188
Weight for length Z-score^c^	1019	− 0.01 (0.02)	0.624	859	0.01 (0.01)	0.197
Head circumference (cm)^d^	568	− 0.00 (0.02)	0.977	461	0.01 (0.02)	0.786
	n	OR (95% CI)		n	OR (95% CI)	
Small for gestational age^e^	1001	1.01 (0.96–1.06)	–	915	1.00 (0.94–1.05)	–
Preterm^f^	1403	1.03 (0.99–1.06)	–	1018	1.06 (1.00–1.12)	–

Models adjusted for

^a^Subcounty, CC, Maternal age, Gestational age, MUAC, Wealth Index, Maternal education, Maternal Height, Infant’s sex

^b^Subcounty, CC, Maternal age, Gestational age, MUAC, Wealth Index, Maternal education, Maternal Height, Infant’s sex, vitamin A consumption (animal & plant)

^c^Subcounty, CC, Maternal age, Gestational age, MUAC, HFIAS, Maternal education, Maternal Height, vitamin A consumption (plant)

^d^Subcounty, CC, Maternal age, Gestational age, MUAC, Wealth Index, Maternal education, Maternal Height (cat), vitamin A consumption (plant), iron suppl freq., MDD-W

^e^Subcounty, CC, Maternal age, MUAC, Wealth Index, Maternal education, Maternal Height, infant’s sex, iron suppl freq

^f^Subcounty, CC, Maternal age, MUAC, Maternal education, Maternal Height, iron suppl freq

^#^Serum ferritin and sTFR were log (ln) transformed and adjusted for inflammation using BRINDA’s Regression Coefficient approach

^$^Body Iron Stores were calculated with the formula − [log(sTFR/ferritin ratio) − 2.8229]/0.1207 (Cook et al., 2003) using values adjusted for inflammation with the BRINDA Regression Coefficient approach

*Significant at *p* < 0.05

**Table 6:** Bivariate and multivariable analyses of maternal RBP and birth outcomes

RBP (µmol/L) & birth outcomes						
	Unadjusted model				Adjusted model	
	n	*β* estimate (SE)	*p* value	n	*β* estimate (SE)	*p* value
Birth weight (kg)^a^	1244	0.01 (0.02)	0.485	1077	− 0.01 (0.02)	0.576
Weight-for-age Z-score^a^	1240	0.01 (0.04)	0.759	1076	− 0.03 (0.05)	0.523
Length-for-age Z-score^b^	1240	0.16 (0.07)*	0.014*	1072	0.12 (0.06)*	0.030
Weight for length Z-score^c^	1020	− 0.06 (0.07)	0.413	867	− 0.02 (0.06)	0.709
Head circumference (cm)^d^	569	0.09 (0.09)	0.329	461	− 0.01 (0.08)	0.864
	n	OR (95% CI)		n	OR (95% CI)	
Small for gestational age (days)^e^	1001	1.19 (0.95–1.50)	–	915	1.25 (0.90–1.74)	–
Preterm^f^	1403	0.83 (0.70–0.98)*	–	1018	0.88 (0.71–1.10)	–

a–f Models were adjusted with the same variables described in Table 5, per birth outcome

*Significant at *p* < 0.05

Maternal iron status (ferritin and BIS) and anemia (hemoglobin) were not significantly associated with any of the assessed birth outcomes. However, all things considered, higher sTFR levels, indicative of iron deficiency, were associated with a 33% decrease in the odds of delivering a preterm baby (AOR: 0.67; 95% CI 0.48–0.94). We further tested for influential points using Cook’s distance and identified 75 observations that may have an effect on the relationship between sTFR and preterm birth. Upon omitting them, the significant relationship remained significant (AOR: 0.50; 95% CI 0.32–0.78).

**Fig. 2:**
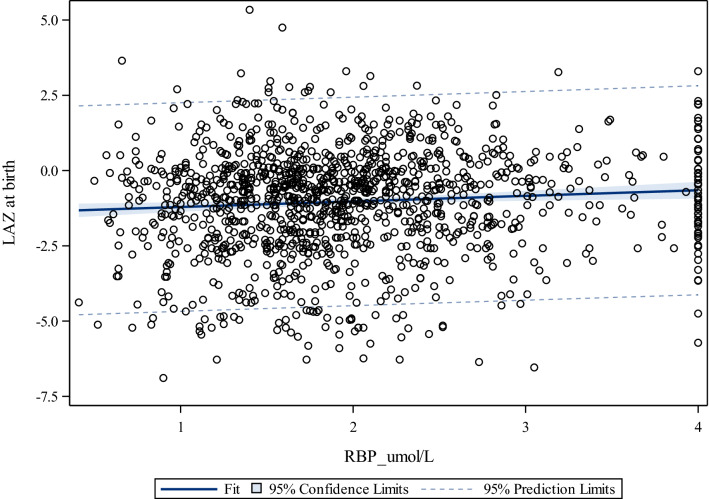
Correlation between maternal RBP (µmol/L) and LAZ at birth, for women in rural Uganda

**Fig. 3:**
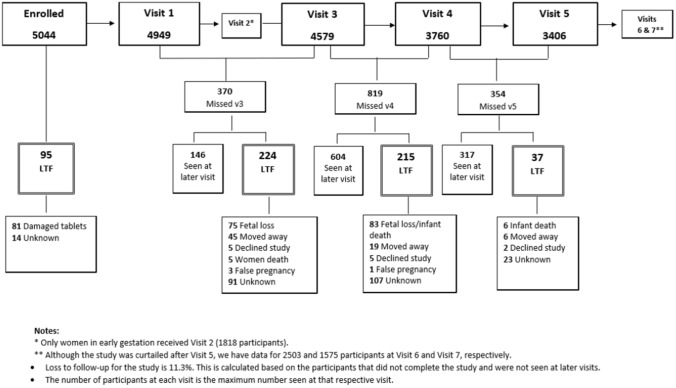
Flowchart of participants in the UBC Study

Regarding vitamin A status (Table 6), we observed a significant positive association between maternal RBP and LAZ (β = 0.12, *p* < 0.030). The bivariate relationship and its magnitude is shown in Fig. 2 (ρ: 0.08 *p* < 0.0046).

## Discussion

This study found no relationship between ferritin or BIS and birth outcomes, but it did observe an association of lower risk of preterm births and higher levels of sTFR, an indicator of iron deficient erythropoiesis. These results are in line with recent findings in China, Papua New Guinea and Taiwan (Fowkes et al., 2018; Hsu et al., 2013; Yuan et al., 2019). Dewey and Oaks have noted that both extreme excess or deficit of iron could lead to risks associated with changes in oxidative stress leading to damaged placental cells, higher blood viscosity through impaired hepcidin regulation, or impaired systemic response to infection/inflammation (Dewey & Oaks, 2017). However, this U-shaped distribution of iron levels was not seen in our population. We speculate that women delivering full-term births were slightly more iron depleted than those delivering preterm, as measured by the higher circulating levels of sTFR. There is evidence of a greater need for iron in the third trimester and closer to term gestation, with women requiring an additional 7.5 mg/day of iron (Col Madendag et al., 2019). sTFR, however, has proved to increase during the third trimester only mildly in iron-replete women but substantially in women with IDA. This indicates that sTFR concentrations are not influenced by pregnancy and are good indicators of iron deficiency (Fisher & Nemeth, 2017).

Increased RBP in mothers was associated with increased LAZ in infants. This finding is consistent with the already established knowledge that vitamin A plays a vital role in full-term gestation by providing fetal reserves and maintaining maternal metabolism during pregnancy (Sapin et al., 2000). Moreover, vitamin A is involved in developing embryonic organ systems, including bone formation (Wiseman et al., 2017). Newborn femur length was increased after maternal treatment with vitamin A during pregnancy (Zhang et al., 2012). However, it is important to note that the WHO recommends routine vitamin A supplementation during pregnancy or lactation only in areas with endemic deficiency since an excess could be teratogenic to the fetus (McCauley et al., 2015).

Regarding prevalence of micronutrient deficiencies, our results showed a relatively low prevalence of vitamin A deficiency in Ugandan women, but that differed depending on the cut-off used. It is worth noting, however, that RBP could possibly be less saturated with retinol due to the normal hemodilution during pregnancy adding uncertainty or underestimating the prevalence estimates when using the standard general cut-off (Sapin et al., 2000; WHO, 2011c). The prevalence of iron deficiency using ferritin was in line with what was previously reported in another study performed in pregnant women in Uganda (Baingana et al., 2015). However, using sTFR, they reported a prevalence of 6.6% versus 22% found in our study (adjusted for inflammation).

Correcting the micronutrients for inflammation using the BRINDA approach yielded a more robust prevalence considering the high prevalence of infection/inflammation in this population. A possible explanation for the low prevalence of vitamin A deficiency observed, is that in adults, especially pregnant women, neither serum RBP nor retinol are good reflections of vitamin A deficiency. This is because serum retinol is controlled homeostatically by the liver stores and it only drops when liver reserves are very low (McCauley et al., 2015; Tanumihardjo et al., 2016).

The low malaria prevalence (6%) found in this study could be explained by the high percentage of IPT use (82%). This treatment, accompanied by other preventive interventions, is a product of national and international programs to reduce malaria in Uganda over the past decade. The prevalence of anemia (14%) was also lower compared to that reported in another study (29%) (Baingana et al., 2015). This decreased anemia prevalence could be explained by the high reported number of iron supplementation during pregnancy.

This study had a number of strengths and limitations. Among the strengths is the use of several biomarkers to assess the same micronutrient levels. In addition to ferritin, sTFR and BIS were measured and calculated for the assessment of maternal iron status, being one of the few studies that included all of them (Dewey & Oaks, 2017). Furthermore, these biomarkers were corrected for the possible effect of inflammation or infection. Additionally, this substudy was part of a large cohort which measured data on several aspects of Ugandan mothers and infants, having a rich set of variables to adjust for in our models. Lastly, various birth outcomes were assessed for each child and measurements were repeated thrice on each participant to minimize measurement errors.

There are several caveats to the findings presented. Firstly, this study is conducted on a subset of the population and may therefore not be reflective of the true status of all the study participants who came from two different parts of the country. We argue against that since the subsample was randomly selected from the overall study population and there was equal representation across the geographic areas included in the study. Additionally, even though these analyses were based on a longitudinal cohort, our substudy was cross-sectional in design and has the disadvantage of not capturing variations in time.

Another limitation could be bias generated due to errors in obtaining birth weights and lengths. These possible errors were addressed by ensuring that only those whose birth weights were obtained within 72 h of birth were included in the analysis. With respect to birth lengths, we included only those infants that had lengths that were considered physiologically plausible. Lastly, we did not collect data on diabetes, hypertension or smoking during pregnancy, all of which are well-known factors associated with adverse birth outcomes.

Future studies will need to build on these results and the literature about the effects of maternal iron and vitamin A with birth outcomes in addition to their possible longer term effects on infants’ growth. Ideally, they should replicate the use of several types of biomarkers to help understand their complexity besides accounting for the possible presence of inflammation.

In summary, these findings indicate that the relationship between maternal iron status during pregnancy and birth outcomes is not linear and needs to be further investigated. This relationship seems to depend on the biomarker used: using serum ferritin or BIS, the results were not significant but with sTFR there was a significant association on preterm births. Regarding vitamin A, our findings additionally confirm that higher, but not excessive, maternal RBP levels during pregnancy could be beneficial for birth outcomes such as LAZ.

### Electronic supplementary material

Below is the link to the electronic supplementary material.

Supplementary file1 (DTA 956 KB)

## Data Availability

The datasets generated and/or analyzed during the current study will be publicly available and are also accessible from the corresponding author upon reasonable request.

## Appendix

See Fig. 3 and Table 7.

**Table 7:** Number of births per subcounty from UBC Study subsample study (n = 1244)

Subcounty	Number of births	Percent
Aduku	96	7.72
Agoro	114	9.16
Agweng	123	9.89
Apac	56	4.50
Atanga	59	4.74
Atyak	73	5.87
Ayer	48	3.86
Bugangari	50	4.02
Buyanja	39	3.14
Bwizi	60	4.82
Kebisoni	73	5.87
Kibiito	85	6.83
Nyamweru	135	10.85
Parombo	88	7.07
Rugyeyo	46	3.70
Ruhija	99	7.96
Total	1244	100

## Abbreviations

AGP: α-1 Acid Glycoprotein
BIS: Body iron stores
BMI: Body mass index
BRINDA: Biomarkers reflecting inflammation and nutritional determinants of anemia
CC: Community connector
CDC: Disease control and prevention
CRP: C-reactive protein
ELISA: Enzyme-linked immunosorbent assay
FER: Ferritin
HFIAS: Household food insecurity and access scale
HGB: Hemoglobin
IDA: Iron deficiency anemia
IPT: Intermittent preventive treatment
LAZ: Length for age z-score
LBW: Low birth weight
MDD-W: Minimum dietary diversity for women
MUAC: Mid-upper arm circumference
RBP: Retinol binding protein
SGA: Small-for-gestational-age
sTFR: Soluble transferrin receptor
UBC: Uganda birth cohort
VHTs: Village health teams
WAZ: Weight-for-age z- score
WHO: World health organization
WLZ: Weight-for-length z-score
